# Using a machine learning approach to identify key prognostic molecules for esophageal squamous cell carcinoma

**DOI:** 10.1186/s12885-021-08647-1

**Published:** 2021-08-09

**Authors:** Meng-Xiang Li, Xiao-Meng Sun, Wei-Gang Cheng, Hao-Jie Ruan, Ke Liu, Pan Chen, Hai-Jun Xu, She-Gan Gao, Xiao-Shan Feng, Yi-Jun Qi

**Affiliations:** 1 School of Information Engineering of Henan University of Science and Technology, 263 Kaiyuan Road, Luolong Qu, Luoyang, 471023 P. R. China; 2 Henan Key Laboratory of Microbiome and Esophageal Cancer Prevention and Treatment; Henan Key Laboratory of Cancer Epigenetics, Cancer Hospital, The First Affiliated Hospital, College of Clinical Medicine, Medical College of Henan University of Science and Technology, 24 Jinghua Road, Jianxi Qu, Luoyang, 471003 P. R. China; 3 The Sixth People’s Hospital of Luoyang, Oncology Department, 14 Xiyuan Road, Jianxi Qu, Luoyang, 471003 P. R. China; 4 Department of Thyroid and Breast Cancer Surgery, The First Affiliated Hospital, College of Clinical Medicine, Medical College of Henan University of Science and Technology, 24 Jinghua Road, Jianxi Qu, Luoyang, 471003 P. R. China

**Keywords:** Esophageal squamous cell carcinoma, Stratifin, Machine learning, Support vector machine, Random forest, Logical regression, Artificial neural network, eXtreme gradient boosting

## Abstract

**Background:**

A plethora of prognostic biomarkers for esophageal squamous cell carcinoma (ESCC) that have hitherto been reported are challenged with low reproducibility due to high molecular heterogeneity of ESCC. The purpose of this study was to identify the optimal biomarkers for ESCC using machine learning algorithms.

**Methods:**

Biomarkers related to clinical survival, recurrence or therapeutic response of patients with ESCC were determined through literature database searching. Forty-eight biomarkers linked to recurrence or prognosis of ESCC were used to construct a molecular interaction network based on NetBox and then to identify the functional modules. Publicably available mRNA transcriptome data of ESCC downloaded from Gene Expression Omnibus (GEO) and The Cancer Genome Atlas (TCGA) datasets included GSE53625 and TCGA-ESCC. Five machine learning algorithms, including logical regression (LR), support vector machine (SVM), artificial neural network (ANN), random forest (RF) and XGBoost, were used to develop classifiers for prognostic classification for feature selection. The area under ROC curve (AUC) was used to evaluate the performance of the prognostic classifiers. The importances of identified molecules were ranked by their occurrence frequencies in the prognostic classifiers. Kaplan-Meier survival analysis and log-rank test were performed to determine the statistical significance of overall survival.

**Results:**

A total of 48 clinically proven molecules associated with ESCC progression were used to construct a molecular interaction network with 3 functional modules comprising 17 component molecules. The 131,071 prognostic classifiers using these 17 molecules were built for each machine learning algorithm. Using the occurrence frequencies in the prognostic classifiers with AUCs greater than the mean value of all 131,071 AUCs to rank importances of these 17 molecules, stratifin encoded by SFN was identified as the optimal prognostic biomarker for ESCC, whose performance was further validated in another 2 independent cohorts.

**Conclusion:**

The occurrence frequencies across various feature selection approaches reflect the degree of clinical importance and stratifin is an optimal prognostic biomarker for ESCC.

## Background

There are approximate 572,000 new cases of esophageal cancer (EC) worldwide in 2018, half of which arise in China [1, 2]. EC ranks sixth and fourth in the incidence and mortality of malignant tumors in China, respectively [3, 4]. The predominant histological subtypes of EC comprise esophageal squamous cell carcinoma (ESCC) and esophageal adenocarcinoma (EAC), among which ESCC accounting for at least 90% of EC in China [5, 6]. Epidemiological studies show that the risk factors of ESCC implicate cigarette smoking, genetic family history, nutritional deficiencies, pickled vegetables intake, hot food and beverage, low socioeconomic status, etc. [7, 8]. In sharp contrast, the increasing risk for EAC is associated with excess body weight and gastroesophageal reflux disorders, which are prevalent in western countries. Furthermore, heavy smoking contributes to an elevated risk of both ESCC and EAC. In the case of alcohol consumption, however, modest to moderate consumption is linked to a reduced risk in ESCC in China, and in EAC in western countries [9]. Heavy alcohol consumption is a strong and well-established risk factor for ESCC in western settings, and cigarette smoking plays a negligible role in ESCC etiology in a high-incidence area of China [8].

As such, it is not possible to distinguish ESCC patients with disparate clinical outcomes under the same exposure conditions based on the risk factors alone. On the other hand, “omics” studies are characterized by poor reproducibility, which could be ascribed to molecular heterogeneity, sample source, tissue processing, detection technique, data analysis, etc. Van’t Veer et al. [10] from Netherlands and Wang et al. [11] from USA analyzed the differentially expressed genes in 295 and 286 cases with breast cancer using gene chip technology, respectively, from which the 70- and 76-signature gene sets for prognostic prediction were developed but with only 3 overlapping genes. Each performed well on its own dataset but not on other datasets. This was also the case for colorectal cancer [12]. It is well-accepted that tumor heterogeneity increases the risk of recurrence and metastasis of tumor patients after treatment and even lead to the resistance to multimodality treatment [13, 14]. Recently, Lin et al. have revealed the molecular heterogeneity of ESCC and its biological significance for tumor development and metastasis from multiple cancers, and revealed the impacts of molecular heterogeneity on the occurrence, development, and prognosis of ESCC [15].

Machine learning is an important branch of artificial intelligence (AI), which provides a possible solution to the current problem of poor reproducibility in group learning. Generally, the machine learning algorithms are divided into weak classifier algorithm and strong classifier algorithm, such as logical regression (LR), support vector machine (SVM) and artificial neural network (ANN) as weak classifier algorithms, and random forest (RF) and eXtreme Gradient Boosting (XGBoost) as strong classifier algorithms. Machine learning algorithms have been widely used in medical science, especially in the diagnosis, prognostic prediction of patients with cancer. For example, Xu et al. identified 5 features among 31 features closely related to the prognosis of ESCC using the genetic algorithm, and established a new ESCC staging system MASAN, showing better prognostic prediction accuracy compared with the currently used TNM staging system [16]. In a prospective cohort study, four machine learning methods, including RF, LR, gradient lifting tree, and ANN, were employed to predict the risk of cardiovascular disease, and the performances were compared between machine learning algorithm and traditional method of ACC/AHA10 annual risk prediction model. The performance of the four machine learning algorithm models was superior [17].

Given the molecular heterogeneity of cancers, we hypothesized that key molecules could serve as genuine prognostic factors even in complicated interactions with other molecules. To further identify key prognostic biomarkers for ESCC, 48 clinically proven molecules associated with ESCC progression were used for subnetwork construction. Using all combinations of 17 component molecules from 3 functional modules, 5 different machine learning algorithms, including LR, SVM, ANN, RF and XGBoost, were used to develop prognostic classifiers. The importances of these 17 molecules were gauged according to the occurrence frequencies in the prognostic classifiers. The prognostic value of stratifin was validated in another 2 independent ESCC cohorts.

## Methods

### Literature search

Literatures related to the prognosis and treatment response of ESCC were retrieved from NCBI PubMed, Web of Science and Embase databases, published up to 31 December 2018, by two independent researchers. The key words for literature searching included “esophageal squamous cell cancer”, “prognosis or recurrence or resistance or sensitivity” and “chemotherapy or chemoradiotherapy”. All relevant studies were retrieved.

### Inclusion and exclusion criteria

We selected the studies using the following criteria: (1) clinical prognosis of patients with ESCC; (2) prediction of clinical response to chemotherapy or chemoradiotherapy; (3) clinical recurrence of ESCC; (4) retrospective and prospective cohort studies; (5) studies published in English. When disagreements occurred between reviewers, a third reviewer was invited for discussion of the eligibility of related studies.

### Datasets downloads

Publicably available mRNA transcriptome data of ESCC from Gene Expression Omnibus (GEO) and The Cancer Genome Atlas (TCGA) datasets included GSE53625 and TCGA-ESCC. GSE53625 included 179 patients with ESCC that were randomly divided into a training cohort of 134 patients and a test cohort of 45 patients. Since the GSE53625 data had been normalized in the original study [18] and all samples in the data set were paired samples, the difference between the expression values of cancer tissue and corresponding adjacent tissue was taken as the input data for all subsequent calculations. TCGA-ESCC contained 82 patients with ESCC, of which 37 Vietnamese patients with ESCC were used for an independent validation.

### Patients and clinical samples

Eighty-six fresh-frozen ESCC with matched noncancerous mucosa samples were collected from the First Affiliated Hospital of Henan University of Science and Technology between 2012 and 2017. All ESCC patients received curative esophagectomy without preoperative neoadjuvant chemoradiotherapy.

### Subnetwork construction

In this study, 48 molecules related to prognosis of ESCC were mapped and imported to NetBox (https://cbio.mskcc.org/tools/netbox/) to establish a molecular interaction subnetwork for network analysis [19]. NetBox, a java-based software tool, integrates four databases including the Human Protein Reference Database (HPRD), Reactome, NCI-Nature Pathway Interaction (PID) Database, and the MSKCC Cancer Cell Map. The shortest path between molecules in the network was defined as 1, denoting that molecules with direct interaction were selected as nodes of the subnetwork. Functional modules in the network were identified and degree of nodes were calculated by igraph R package.

### Introduction of machine learning algorithms

This study used 5 machine learning algorithms, including LR, SVM, ANN, RF and XGBoost, to develop classifiers for prognostic classification.

The LR model is a generalized linear model, which is based on linear regression with a layer of Sigmoid function mapping. LR regression model is one of the most commonly used methods in medical research [20, 21].

SVM is a supervised learning method developed by Cortes and Vapnik in 1995 [22]. The support vectors are used to find the best hyperplane and then classify samples with different labels. The nonlinear features are mapped to the new high dimensional space by constructing a mapping function, and the inner product operation in the mapping space is simplified by kernel function to ensure that the results were equivalent, to achieve the linear separability of the samples. In this study, the Radial Basis Function (RBF) kernel function was used, and the RBF’s transformation method was as follows:

$K\left(x,{x}^{\prime}\right)=\exp \left(-\frac{{\left\Vert x-{x}^{\prime}\right\Vert}^2}{2{\sigma}^2}\right)$, where *σ* is the hyper-parameter controlled in accordance with deviation and error of variance.

Neural networks are an important machine learning technology and have widespread applications with advances of scientific computing capabilities such as supercomputers and quantum computing. In general, a neural network consists of an input layer, multiple hidden layers, and an output layer. The most important element in a neural network is the design of hidden layer and connection weight between neurons. Logistic regression belongs to the neural network with zero hidden layers.

RF and XGBoost are two integrated learning algorithms based on bagging and boosting algorithms, respectively. Integrated learning uses a certain method to learn multiple weak classifiers with some differences followed by combination of these classifiers. If the error rate of weak classifier is less than 0.5, the combination of multiple weak classifiers will gradually increase predictive ability and reduce classification error to achieve classification.

### Development of classifiers

For 179 patients with ESCC samples, labels were assigned according to the survival time. Label 1 denotes the ESCC cases with survival times of more than 3 years and the remaining cases were labeled as 0. In the training cohort, cross-validation and parameter optimization were used to develop the models, and the test cohort was used for validation. Receiver operating characteristic (ROC) curve analysis was used to estimate predictive values of machine learning classifiers and the area under the curve AUC (area under ROC Curve) was calculated.

For each machine learning algorithm, 131,071 models representing various combinations of 17 selected features were established, and AUCs of the models in training and test cohort were calculated. During the development of classifiers, candidate classifiers were those classifiers with AUCs greater than the average of AUCs across all classifiers. Among all candidate classifiers, top 1000 models with the highest AUC values in test cohort were selected, and the occurrence frequencies of each molecule were counted in these 1000 classifiers. Top 5 molecules with the highest occurrence frequency were regarded as the important molecules of the corresponding machine learning algorithm.

The construction and testing of the classifiers in this study were implemented by using R 3.6.3. The weak classifier uses R packages such as bestglm, e1071, and nnet, and the integrated learning algorithm uses random forest and xgboost.

### RNA extraction and quantitative RT-PCR

Total RNA of 86 pairs of ESCC samples with matched noncancerous tissues were isolated using Trizol reagent (Invitrogen, Carisbad, CA), and reverse transcription was performed using 1 μg of total RNA (Promega, USA). The primer pair for stratifin was as follows: forward primer, 5′-GACTACTACCGCTACCTGGC-3′, and reverse primer, 5′-GTTGGCGATCTCGTAGTGGA-3′. GAPDH was used as an internal standard and its primer pair was as follows, forward primer, 5′- GCCACATCGCTCAGACACC − 3′, and reverse primer, 5′- GATGGCAACAATATCCACTTTACC − 3′. Quantitative RT-PCR was performed in triplicate on an Applied Biosystems 7900 quantitative PCR system (Foster City, CA, USA). The Ct values were used for comparison using 2^-ΔΔCt^ method with GAPDH as the internal standard.

### Statistical analysis

Differences of the quantitative data between 2 groups were performed using the unpaired or paired Student t-test. The relationship between the abundance of western blot and the expression level of SFN was analyzed by using linear regression. Overall survival was calculated from the date of surgery to the date of last follow-up or death. The Kaplan-Meier survival curves and log-rank tests were performed to determine the statistical significance of overall survival. All tests were 2-tailed and *P* < 0.05 were designated as significantly different.

## Results

### Prognostic biomarkers of esophageal squamous cell carcinoma

We initially retrieved 38 articles, which reported a total of 48 molecules associated with the clinical survival, recurrence or therapeutic outcome of ESCC patients (Table 1). In addition, a long non-coding RNAs LOC285194 and 6 microRNAs, including miR-23a, miR-24, miR-382, miR-7, and a combination of miR-133a and miR-133b, were identified as well. Due to their low numbers, these microRNAs and long non-coding RNA were excluded from this study. Thus, 48 unique molecules were included for subsequent study.

**Table 1:** Thirty-eight studies reporting 48 molecules that were associated with clinical survival, recurrence or therapeutic outcome of ESCC patients

Biomarkers	Prognosis/recurrence /therapeutic outcome	PMID	Sample sizes	Conclusions
BRCA1	Prognosis and chemoradiotherapy	23,326,344	144	Low BRCA1 expression was an independent prognostic factor in cisplatin-based chemotherapy (HR 0.29, 95% CI 0.12–0.71; *P* = 0.007) or chemoradiotherapy (HR 0.12, 95% CI 0.04–0.37; *P* < 0.001) group.
CCNA2	Chemotherapy	23,205,070	48	The expression of cyclin A was an independent prognosis factor in patients with ESCC following paclitaxel-based chemotherapy.
CCND1	Prognosis and radiochemotherapy	9,988,238	172	Patients with cyclin D1-positive carcinomas showed significantly worse overall survival than patients with cyclin D1-negative carcinomas (HR 2.14, 95% CI 1.134–3.42; *P* = 0.0038)
CD163 & CD68	Prognosis and neoadjuvant chemotherapy	25,752,960	210	High infiltration of CD68+ macrophages and CD163+ macrophages was significantly associated with poor prognosis for patients undergoing neoadjuvant chemotherapy (*P* = 0.057, *P* = 0.003).
CD274	Prognosis and chemoradiotherapy	26,623,522	45	The higher PD-L1 H-scores had poorer overall survival (median 16.7 versus 32.9 months, P = 0.02) than those with lower H-scores. (HR 2.29, 95% CI 1.12–4.69; *P* = 0.023)
CD44 & PROM1	Prognosis	27,748,881	47	Patients with strong expression of CD44 or CD133 and those with a high ratio of CD133-positive tumor cells showed significantly poor prognosis regardless of the effect of chemotherapy. PROM1 (HR 5.05, 95% CI 1.12–4.69; P = 0.023)
CDKN2A	neoadjuvant chemotherapy	26,514,506	101	ESCC tumors that were found positive for p16 expression appeared to fall into responders group rather than non responders (*P* = 0.008) and reported with less mortality (*P* = 0.048).
CEA & KRT19	Chemoradiotherapy	19,863,186	84	CEA may be helpful in predicting the responsiveness in ESCC of primary lesions to CRT, with the effective rates (CR + PR) in CEA high and low groups of 58.3% (14/24) and 93.3% (56/60), respectively (*P* = 0.013 and 0.013).
EGFR	Chemoradiotherapy	17,940,077	62	The difference in the CR rate between EGFR positive and -negative groups was significant (CR rate: 62% vs. 34%; *P* = 0.037).
CRP	Chemoradiotherapy	21,224,533	36	Serum CRP can predict of CRT response with an accuracy of 75%.
ERCC1	Chemotherapy	23,263,828	46	Patients with ERCC1 negative tumors had a higher treatment response than the ERCC1 positive group (radiological response rates; 92.3% vs.50%, *P* = 0.013).
FAM84B	Neoadjuvant chemoradiation	25,980,316	21	The fold-change of circulating FAM84B mRNA expression can predict the pCR with an AUC of 0.73.
FDXR	Prognosis and Neoadjuvant chemoradiation	26,637,858	50	Fdxr was significantly correlated with postoperative outcomes and an independent prognostic factor (HR 4.950, 95% CI 1.603–15.38; 0.012).
HOXC6 & HOXC8	Prognosis	24,525,058	274	HOXC6 and HOXC8 were independent prognostic factors in patients with ESCC. HOXC6: (HR 1.341, 95% CI 0.895–2.010; *P* = 0.045); HOXC8: (HR 1.657, 95% CI 1.146–2.395; *P* = 0.007).
IL6R	Prognosis	23,648,090	218	The sIL6R level was one of several significant independent predictors of an unfavorable outcome. (HR 3.20, 95% CI 1.34–7.53; *P* = 0.008)
MDM2 & MKI67	Prognosis and chemoradiotherapy	25,880,782	79	MDM2 and p16 are predictive markers for chemoradioresistance in cStageIII ESCC and Ki-67 is a prognostic marker following dCRT in cStageIII ESCC.
MLH1	Prognosis	18,053,639	51	The expression of hMLH1 is a potential marker of tumor response and survival.
MMS19	Chemoradiotherapy	25,892,874	103	High cytoplasmic MMS19 expression was associated with a good response to chemoradiotherapy (OR 11.5, 95% CI: 3.0–44.5; P < 0.001).
MT3	Prognosis	16,351,731	64	Esophageal squamous cell carcinomas with negative p53, positive CDC25B, and negative MT expressions respond well to CRT.
MUC13 & MUC20	Prognosis and neoadjuvant chemotherapy	26,323,930	186	The median survival time of patients with low MUC13/high MUC20 expression was significantly shorter than that of patients with high MUC13/low MUC20 expression (27.7 months vs. 59.5 months, *P* = 0.021; HR 0.531, 95% CI: 0.299–0.944; *P* = 0.031).
MUC4	neoadjuvant chemotherapy	26,673,820	186	Low expression of MUC4 and MUC20 in resection samples was significantly correlated with better TRG (tumor regression grade). MUC4 and MUC20 were identified as potential biomarkers for predicting the efficacy of neoadjuvant chemotherapy in ESCC patients.
NOTCH1 & PIK3CA	Prognosis and Chemotherapy	26,528,858	104	NOTCH1 mutations was correlated with shorter survival times and failed to respond to chemotherapy, whereas PIK3CA mutations pointed to better responses to chemotherapy and longer survival times than patients without PIK3CA mutations.
PTGS2	Prognosis and Chemoradiotherapy	21,437,756	58	Negative or weak expression of PTGS2 was correlated significantly with CRT response (OR 6.296, 95% CI 1.58–25.096; *P* = 0.010).
PTPN6	Prognosis	32,536,826	184	Elevated PTPN6 expression indicated longer OS (HR 1.123, 95% CI: 0.565–2.230; *P* = 0.741).
RAD51	Prognosis and recurrence	24,065,387	89	Rad51 expression in ESCC was associated with poor survival (*P* = 0.0324) and recurrence (*P* = 0.0171).
REG1A	Prognosis	23,645,481	177	REG1A expression was a significant prognostic factor (HR 3.095, 95% CI: 1.569–5.943; *P* = 0.0015).
SFN	Chemoradiation therapy	15,999,354	62	SFN-positive expressions were closely related to the response to CRT.
	Prognosis	24,743,601	278	Downregulation of 14–3-3σpredicts poor survival, suggesting that 14–3-3σmay be a biomarker for early detection of high-risk subjects and diagnosis of ESCC. (HR 0.466, 95% CI 0.251–0.866; *P* = 0.016).
	Prognosis	20,108,042	148	Reduced stratifin expression, T4 stage, lymph node metastasis, and distant metastasis were independent risk factors for worse prognosis in ESCC patients.
SGTA	Prognosis	23,939,810	120	SGTA expression indicated poor prognosis (RR 3.513, 95% CI: 2.161–9.791; P = 0.016).
TGFB1 & VEGFA	Prognosis	24,623,035	79	VEGFA and TGFB1 were significantly associated with pathological response and/or DFS, and may be used to predict pathological response and survivals for ESCC patients receiving combined modality therapy.
TP53 & RRM2B	Prognosis and chemoradiotherapy	15,655,547	62	p53 or p53R2 (RRM2B) expression was correlated with a favorable response to CRT (*P* = 0.0001 or 0.041 clinical, P = 0.016 or 0.0018 histological, respectively; TP53, RR 2.688, 95% CI: 1.157–6.250; *P* = 0.0011. RRM2B, RR 2.469, 95% CI: 1.164–5.235; *P* = 0.0057).
	Prognosis	25,135,238	36	The median tumor associated survival was 34.2 months for patients with normal TP53, compared with 8.9 months for those with mutant TP53. The latter had a 3-fold higher risk of death (HR 3.01, 95% CI 1.359–6.86; *P* = 0.005).
	Prognosis	10,414,702	42	The current study indicated that p53 mutation of tumor tissues might be a prognostic factor for esophageal squamous cell carcinoma cases and one of the risk factors for its recurrence.
	Chemotherapy	19,941,080	97	Patients with mutations in p53 therefore showed significantly poorer prognosis than those without mutant p53.
RAC3 & TRAM1	Prognosis and chemoradiotherapy	19,552,757	98	Overexpression of AIB1/RAC3/ TRAM1 is a useful predictor of CRT resistance and an independent molecular marker of poor prognosis for ESCC patients.
ALDH1A1, ALDH1A2, ALDH1A3, ALDH1B1, ALDH1L1, ALDH1L2	Prognosis and recurrence	22,847,125	152	ALDH1 was a predictor of postoperative recurrence and prognosis in ESCC, and CD44 might be a predictor of recurrence and prognosis.
PITX2	Prognosis and chemoradiotherapy	23,132,660	454	High expression of PITX2 was associated with poor disease-specific survival (HR 1.732, 95% CI 1.133–2.646; *P* = 0.011) in ESCC.

### Identification of key prognostic molecules

**Fig. 1:**
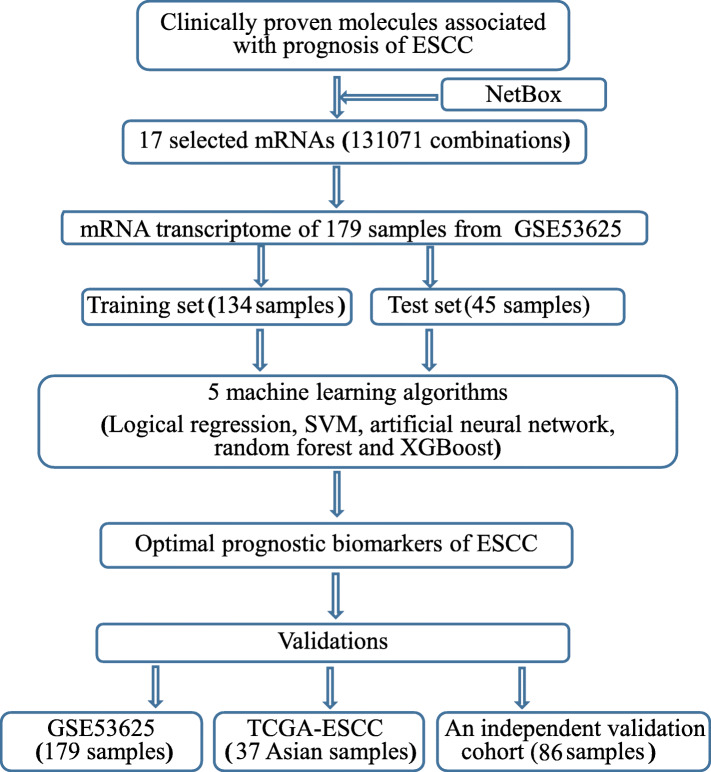
The flowchart for identification of optimal prognostic molecules for esophageal squamous cell carcinoma

**Fig. 2:**
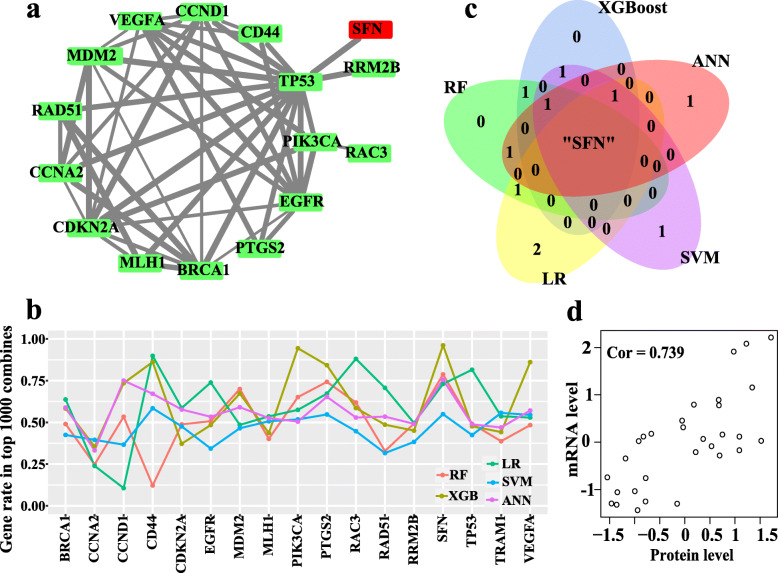
NetBox and Machine learning model. **a** Molecular interaction network constructed by NetBox and STRING; **b** The occurrence frequencies of each molecule in top 1000 classifiers across 5 machine learning algorithms; **c** The intersection of the optimal genes from five machine learning algorithms; **d** Pearson correlation analysis between protein and mRNA expression levels of SFN detected by WB and RT-PCR, respectively. LR: logical regression; SVM: support vector machine; ANN: artificial neural network; RF: random forest; XGBoost: eXtreme gradient boosting; WB: Western bolt

Our approach for validating clinically proven molecules associated with prognosis of ESCC is summarized in Fig. 1. All 48 molecules were used to construct a protein-protein interaction network using NetBox. The shortest path between the molecules in the network was defined as 1, indicating that those molecules with direct interaction were retained as nodes in the network. This study is based on the local version of Java and Python using NetBox algorithm to define the functional modules. By inputting the Entrez ID of 48 molecules, 3 functional modules containing a total of 17 molecules as vertices and 19 edges were identified. A subnetwork of 16 molecules among these 17 molecules based on STRING database (https://string-db.org/) was built with 0.7 as the minimum interaction score (Fig. 2a).

### Prognostic classification using 5 machine learning algorithms

Seeking to improve the predicative accuracy of ESCC prognosis, 5 different machine learning algorithms, including LR, SVM, ANN, RF and XGBoost, were leveraged for prognostic classification using the 17 prognostic molecules. Among the prognostic models with AUCs greater than the mean value of all AUCs of 131,071 models for each algorithm, the importances of those 17 prognostic molecules were weighted by their occurrence frequencies. Table 2 shows the top 5 important molecules identified by each machine learning algorithm and the intersecting molecule is SFN only (Fig. 2c), indicating that SFN may be the optimal prognostic biomarker for ESCC.

**Table 2:** The top 5 important molecules identified by each machine learning algorithm

Molecule rank	Weak classifiers			Strong classifiers	
	LR	SVM	ANN	RF	XGBoost
1	CD44	CD44	**SFN**	**SFN**	**SFN**
2	RAC3	TRAM1	CCND1	PTGS2	PIK3CA
3	TP53	**SFN**	CD44	MDM2	CD44
4	EGFR	PTGS2	PTGS2	PIK3CA	VEGFA
5	**SFN**	VEGFA	MDM2	RAC3	PTGS2

### Correlation of stratifin mRNA and protein expression

Because we have reported that stratifin protein encoded by SFN by immunohistochemical assay was reduced significantly in ESCC compared with normal esophageal mucosa and intraepithelial neoplasia, the present study, however, revealed that stratifin mRNA expression was downregulated in ESCC compared with noncancerous tissues using an ESCC cohort of GSE53625. We assessed the correlation between stratifin protein and mRNA expression. Figure 2d shows that stratifin protein levels strongly correlate with its mRNA levels in ESCC tissues, detected by Western blot and by RT-PCR, respectively, suggesting that both the protein and mRNA expression patterns of stratifin may have prognostic implication in ESCC.

### Prognostic validation of stratifin

**Fig. 3:**
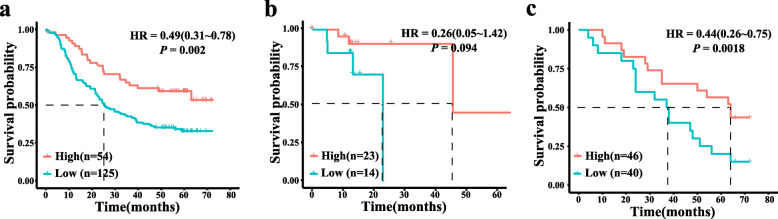
Kaplan-Meier survival curves of ESCC patients in each dataset. **a** Kaplan-Meier survival curves of ESCC patients in GSE53625 dataset; **b** Kaplan-Meier survival curves of ESCC patients in TCGA-ESCC dataset (37 Asian cases); **c** Kaplan-Meier survival curves of ESCC patients in an independent cohort of 86 patients with ESCC

Using the dataset of GSE53625, 125 and 54 patients with ESCC were dichotomized into high-risk and low-risk subgroups according to optimal expression threshold of stratifin. The Kaplan-Meier survival analysis showed that the median survival times of the high-risk and low-risk subgroups were 25.5 months and > 60 months, respectively (Fig. 3a). Moreover, log-rank test showed that the survival times of two groups were significantly different, with a hazard ratio of 0.49 for patients with high stratifin expression (95% CI, 0.31 to 0.78, *P* = 0.002). The 3-year survival rates for these 2 subgroups were 42.4 and 63.1%, respectively. These results indicate that high expression of gene SFN is favorable to long-term survival of ESCC patients. In the 37 cases of ESCC with Asian ancestry from TCGA database, there was a trend for a favorable prognosis in ESCC patients with high mRNA levels of stratifin (*P* = 0.094, Fig. 3b).

We then validated the prognostic value of stratifin mRNA in another independent 86 ESCC cases. Using the median of stratifin mRNA levels as a cut-off value, 40 patients with ESCC were assigned to the high-risk subgroup and the other 46 patients to the low-risk subgroup. In consistent with previous results, ESCC patients in the high-risk subgroup had a significantly poorer survival than those in the low-risk subgroup. The median survival time for patients in the high-risk group was 37.5 months, while that for ESCC patients in the low-risk group was 60 months. The 3-year survival rates for the high-risk and low-risk subgroups were 53.6 and 73.5%, respectively. The log-rank test showed that the survival times of two groups were significantly different, with hazard ratio of 0.44 (95% CI, 0.26 to 0.75, *P* = 0.0018, Fig. 3c).

## Discussion

In this study, 48 molecules associated with clinical outcome of ESCC were used for construction of a molecular interaction network and subsequent identification of functional modules. Afterwards, all combinations of 17 component molecules from 3 modules were used to develop prognostic classifiers with 5 machine learning algorithms. Stratifin encoded by SFN was identified as the key prognostic biomarker for ESCC because it was the top overlapping molecule across the 5 prognostic methods used in this study. The down-regulation of stratifin mRNA and protein expression was associated with an overall poor survival of ESCC patients in 3 independent cohorts. Therefore, stratifin encoded by SFN was a robust biomarker for prognostic prediction of ESCC patients.

A variety of computational methods, such as dimensionality reduction [16], Cox multivariate regression [23], and subnetworks construction [24], have been used to identify biomarkers for detection, diagnosis and prognosis of patients suffering from cancers. In most cases, these methods were applied independently. As a result, distinct sets of molecules are identified by using various algorithms. It is conceivable, however, that the key molecules exerting crucial biological functions in cancer progression might be identified by these different computational analyses. The frequencies of overlapping molecules identified across these computational algorithms represent the degrees of functional importance. Using a subset of 38 miRNAs with experimental evidence associated with breast cancer, Oneeb et al. employed 3 feature selection methods, including Information Gain, Chi Squared, and Least Absolute Shrinkage and Selection Operation, to rank the importances of miRNAs. The top 10 important miRNAs were utilized to build optimal classifiers for discrimination between breast cancer cases and healthy subjects using RF-based and SVM-based algorithms. A 3-miRNA signature showed the best performance for diagnosis of breast cancer, indicating that not all miRNAs are equally important as cancer biomarkers [25]. Notably, these results demonstrate that the machine learning is a useful tool for feature selection without transformation of original features. In the present study, 48 biomarkers with clinical evidence for prognosis of ESCC were used to construct a subnetwork with 3 functional modules, including 17 component molecules. To rank the importances of these 17 molecule features, 5 machine learning algorithms were used for feature selection with SFN as the top overlapping gene, suggesting that SFN might be the optimal prognostic biomarker for ESCC.

In line with our previous findings, the expression pattern of stratifin mRNA resembled its protein expression, both of which were downregulated in ESCC compared with adjacent noncancerous mucosa. In the ESCC cohort of GSE53625, stratifin mRNA was an independent prognostic biomarker. This was also the case in another independent 86 ESCC cohort. Furthermore, a strong positive correlation between mRNA and protein expression of stratifin was found as well. Stratifin, one of the seven isoforms of 14–3-3 proteins in mammals, form homodimers and heterodimers that could bind to a number of target proteins in native state. Through association, stratifin regulates the functions of its ligands, including cytoskeletal dynamics, cell cycle regulation, polarity, adhesion, motility, mitogenic signaling and oncogenic signaling. In response to DNA damage, p53 can induce stratifin expression. In this manner, upregulation of stratifin causes G_2_ arrest through sequestration of cdc2-cyclin B1 complex in cytoplasm and allows the repair of damaged DNA before further cell cycle progression. Thus, stratifin has been suggested to be a potential tumor suppressor. Decreased expression levels of stratifin occur frequently in many human cancers including breast [26–33], lung [34], colon [35], liver [36], prostate [37–39], ovary [40–42], nasopharynx [43], and oral cancers [44]. In addition, downregulation of stratifin in ESCC has been reported in several studies, which showed a negative correlation between SFN and clinical outcome [45–47]. Collectively, the present study provided further evidence supporting stratifin as a reliable prognostic biomarker for ESCC.

There are certain limitations to our study. Firstly, the present study only validated the clinical significance of stratifin in ESCC. Due to tumor heterogeneity, a composite biomarker comprising multiple functional molecules could represent the biology of ESCC much better than single molecule, and thus is able to improve the overall prediction of ESCC outcome. Secondly, liquid biopsy, in particular a simple blood test, offers a less-invasive approach to real-time monitor metastatic progression and therapeutic outcome of ESCC compared with tissue biopsy. The profile of stratifin in blood of ESCC patients should be characterized in future studies.

## Conclusions

The present study presents stratifin as an optimal prognostic biomarker for ESCC using machine learning algorithms. In 3 independent cohorts of ESCC, stratifin can discriminate between ESCC patients with different clinical outcomes. Further prospective studies from different institutions are needed to validate the robustness of stratifin in prognostic prediction of ESCC patients. Thus, our study demonstrates that the overlapping frequencies across different feature selection approaches represent the degree of importance, with top one as the key molecule with clinical implication. This method of mining key molecules that stably affect the prognosis of ESCC could be applied to the other relevant research.

## Data Availability

The data sets of GSE53625 from GEO (https://www.ncbi.nlm.nih.gov/geo/) and 37 ESCC cases with Asian ancestry from TCGA (UCSC Xena, https://xena.ucsc.edu/) are public open and available.

## Abbreviations

EC: Esophageal cancer
ESCC: Esophageal squamous cell carcinoma
EAC: Esophageal adenocarcinoma
GEO: Gene Expression Omnibus
TCGA: The Cancer Genome Atlas
AI: Artificial intelligence
LR: Logical regression
SVM: Support vector machine
ANN: Artificial neural network
RF: Random forest
XGBoost: eXtreme Gradient Boosting
WB: Western blot
PCR: Polymerase chain reaction
ROC: Receiver operating characteristic
AUC: Area under ROC curve
